# Transcriptome analysis during berry development provides insights into co-regulated and altered gene expression between a seeded wine grape variety and its seedless somatic variant

**DOI:** 10.1186/1471-2164-15-1030

**Published:** 2014-11-27

**Authors:** Chinedu Charles Nwafor, Ivana Gribaudo, Anna Schneider, Ron Wehrens, Maria Stella Grando, Laura Costantini

**Affiliations:** Fondazione Edmund Mach, Research and Innovation Centre, Via E. Mach 1-38010 San Michele all’Adige, Trento, Italy; Consiglio Nazionale delle Ricerche, Istituto per la Protezione Sostenibile delle Piante, Unità Grugliasco, Largo P. Braccini 2-10095, Grugliasco, Torino Italy; Dipartimento di Scienze Agrarie, Università degli Studi di Torino, Forestali ed Agroalimentari, Largo P. Braccini 2-10095, Grugliasco, Torino Italy

**Keywords:** *Vitis vinifera*, Seed content, mRNA-Seq, Differential gene expression, Functional category enrichment, Candidate gene

## Abstract

**Background:**

Seedless grapes are greatly appreciated for fresh and dry fruit consumption. Parthenocarpy and stenospermocarpy have been described as the main phenomena responsible for seedlessness in *Vitis vinifera*. However, the key genes underpinning molecular and cellular processes that play a significant role in seed development are not well characterized. To identify important regulators and mechanisms that may be altered in the seedless phenotype, we performed a comprehensive transcriptional analysis to compare the transcriptomes of a popular seeded wine cultivar (wild-type) and its seedless somatic variant (mutant) at three key developmental stages.

**Results:**

The transcriptomes revealed by Illumina mRNA-Seq technology had approximately 98% of grapevine annotated transcripts and about 80% of them were commonly expressed in the two lines. Differential gene expression analysis revealed a total of 1075 differentially expressed genes (DE) in the pairwise comparison of developmental stages, which included DE genes specific to the wild-type background, DE genes specific to the mutant background and DE genes commonly shared in both backgrounds. The analysis of differential expression patterns and functional category enrichment of wild-type and mutant DE genes highlighted significant coordination and enrichment of pollen and ovule developmental pathways. The expression of some selected DE genes was further confirmed by real-time RT-PCR analysis.

**Conclusions:**

This study represents the most comprehensive attempt to characterize the genetic bases of seed formation in grapevine. With a high throughput method, we have shown that a seeded wine grape and its seedless somatic variant are similar in several biological processes. Nevertheless, we could identify an inventory of genes with altered expression in the mutant compared to the wild-type, which may be responsible for the seedless phenotype. The genes located within known genomic regions regulating seed content may be used for the development of molecular tools to assist table grape breeding. Therefore the data reported here have provided a rich genomic resource for practical use and functional characterization of the genes that potentially underpin seedlessness in grapevine.

**Electronic supplementary material:**

The online version of this article (doi:10.1186/1471-2164-15-1030) contains supplementary material, which is available to authorized users.

## Background

Over the past decade there has been a sustained increase in the world production of table grapes, which reached 22.3 million tons [1]. This is largely due to consumer demand for seedless grape for fresh and dry fruit consumption. Nowadays, most breeding programs focus on the generation of new cultivars (cvs) combining seedlessness together with other traits such as large berry size, muscat flavor or crispiness.

*Vitis vinifera* L. is considered a good model for the study of seed development in fruit crops. Two different mechanisms are involved in grape seedlessness, namely parthenocarpy and stenospermocarpy. Usually, in parthenocarpic conditions fruit develops from the ovary in the absence of fertilization yielding small berries that completely lack seeds (e.g. cv Black Corinth), whereas in stenospermocarpy pollination and fertilization take place normally, but seed development aborts at an early stage (2–4 weeks) after fertilization and berry size at harvest is reduced (e.g. cv Sultanina) [2, 3]. Most cultivated seedless grapes exhibit stenospermocarpy. The major events that take place in grapevine normal seed development, parthenocarpy and stenospermocarpy are shown schematically in [4] and are described in detail by [5, 6].

In *Arabidopsis*, genetic studies have revealed several genes that participate in seed development like *SHOOT MERISTEMLESS* (*STM*), *CUP*-*SHAPED COTYLEDON* (*CUC1* and *CUC2*), *AINTEGUMENTA* (*ANT*)*, SPATULA* (*SPT*)*, AGAMOUS* (*AG*) *MADS box genes AG-SHATTERPROOF* (*SHP1* and *SHP2*), *SEEDSTICK* (*STK*, also known as *AGL11*), *NOZZLE/SPOROCYTELESS* (*NZZ/SPL*), *EMBRYO-DEFECTIVE* (*EMB*) and *INO*[7–9], including those that regulate endosperm formation such as *CRINKLY4* and *BET1*[10, 11], embryo differentiation such as *EMBRYO-DEFECTIVE* (*EMB*) and *LEAFY COTYLEDON* (*LEC*) [12–14], and seed coat development such as *APETALA 2* (*AP2*) and *TRANSPARENT TESTA 16* (*TT16*) [15]. Also, molecular studies with *Arabidopsis*, tomatoes, and other plants have revealed *cis*-regulatory elements of several genes active during seed development, mostly the transcription factors (TFs) that play a role in their regulation, i.e. *LEAFY COTYLEDON (LEC)* genes and *AGAMOUS-like 15* (*AGL15*) [16–18]. Nevertheless, in grapevine the identities of most regulators of seed development and their direct targets are largely unknown. To date, a number of studies have adopted QTL (Quantitative Trait Locus) analysis to dissect the genetic determinism of seedlessness [19–23]. A MADS-box ovule identity gene (*VvAGL11*) was proposed as the major positional and functional candidate gene for stenospermocarpy and tested for usefulness in marker-assisted selection [24, 25]. However, very few studies have looked for genes possibly responsible for seedlessness by comparison of gene expression profiles in seeded and seedless grapes. For instance, differential expression analysis in seeded and seedless clones of cv Sultanina by [26, 27] allowed the identification of a chloroplast chaperonin (ch-Cpn21) resulting in seed abortion when silenced in tobacco and tomato, and of a ubiquitin extension protein (S27a) having a probable general role in the control of organ development in grapevine. Recently, differential expression analysis during ovule development in seeded and seedless cultivars identified grape metacaspase genes, consistent with a role of programmed cell death in stenospermocarpy [28].

To identify regulators and processes required for seed development that may be altered in the seedless phenotype, somatic variants are invaluable resources. At the same time an analytical approach that provides a holistic view of the transcriptional landscape during seed development in both phenotypes is equally vital. In grapevine, somatic variation arises from mutation or epimutation events that first occur in a single cell belonging to a specific cell layer. Once at least one shoot apical meristem is colonized by the mutated cell in one or both cell layers, the mutation can be transmitted by bud propagation or eventually sexual reproduction [29]. However, identification of somatic variants in grapevine is a time and labor intensive task, which requires genetic and phenotypic characterization of large germplasm collections [30]. At the same time, the application of deep sequencing techniques to survey the total population of RNA within a tissue has made RNA-Seq a popular and comprehensive approach to deduce and quantify the transcriptome [31]. Its potential has been demonstrated in the *de novo* transcriptome characterization of *Vitis vinifera* cultivars [32] and gene expression profile of grape berry during key developmental stages [33–35].

In this paper, we exploited the availability of a spontaneous seedless somatic variant (hereafter mutant, MT) derived from Sangiovese (hereafter wild-type, WT), a widespread seeded wine cultivar in Italy [30]. This mutant has a gross morphology of vines identical to the wild-type except for absence of seeds, reduced berry and bunch size at harvest. With the aim of understanding the molecular mechanisms driving the seedless phenotype, we analyzed the transcriptional responses possibly related to seed development in the wild-type and the mutant using Illumina mRNA-Seq technology.

## Methods

### Sample collection

Samples were collected from wild-type and mutant plants in the germplasm collection of Grinzane Cavour maintained by CNR-Istituto di Virologia Vegetale di Grugliasco (Torino, Italy).

For molecular marker analysis young leaves were gathered.

To create inventories of gene expression at successive stages of seed formation, three key time points along grape berry development were selected corresponding to stages E-L 15 (single flowers in compact groups), E-L 27 (young berries enlarging) and E-L 38 (berries harvest-ripe) of the modified E-L system described by [36]. Samples were collected for both clones in the following dates: 12th May, 10th June and 16th September 2010. When matched to the number of days from bloom (DFB) shown in [4], these time points could be assigned to two main categories: “before” (E-L 15) and “after” (E-L 27 and 38) fertilization. A detailed description of how sampling dates were matched to DFB is reported in Additional file 1. For each developmental stage two independent samples (biological replicates) were collected. A biological replicate was composed of the whole inflorescence for stage E-L 15 and of the whole bunch for stages E-L 27 and 38.

### Genomic DNA extraction and SSR genotyping of the wild-type and the mutant

Total genomic DNA was extracted from young immature leaves as described by [37]. Fifty-eight SSR (simple sequence repeat) markers, spread across the nineteen chromosomes of grapevine genome, were used to genotype the wild-type and the mutant (Additional file 2). Of this set, twenty SSR markers were previously described by [37], thirty-two SSR markers used by [23] and six SSR markers developed by [24].

PCR amplifications for multiplex panels were carried out in a final volume of 12.5 μl containing 10 ng of genomic DNA, 0.25 mM of each dNTP, 2 mM MgCl_2_, 1.5 U Taq DNA Polymerase (AmpliTaq Gold™, Applied Biosystems, Foster City, CA). The amplification protocol was as follows: 7 min at 95°C; 30 cycles of 45 sec at 95°C, 1 min at 54°C, 30 sec at 72°C; and 1 hour at 72°C. Primers failing to amplify at 54°C were further tested in single panel at different annealing temperatures.

PCR products (0.5 μl) were mixed with 9.3 μl of formamide and 0.2 μl of the GeneScan™ 500 ROX® Size Standard (Applied Biosystems) and 0.5 μl of this mix was subjected to capillary electrophoresis on an ABI PRISM 3130 Genetic Analyzer (Applied Biosystems) to separate DNA fragments. GeneMapper v3.5 (Applied Biosystems) was employed for the allele size estimation.

### RNA extraction

For each sample total RNA extraction was performed from a lot of flowers/berries in triplicate (technical replicates), using the Spectrum™ Plant Total RNA kit (Sigma-Aldrich, St. Louis, MO) following the manufacturer’s protocol. RNA quality and quantity were determined using a Nanodrop 8000 (Thermo Scientific, Wilmington, DE) and a Bioanalyzer 2100 (Agilent, Santa Clara, CA).

### Library preparation and sequencing

For transcriptomic analysis a single biological replicate was used due to economic constraints. Total RNA from the three technical replicates of each sample were pooled for a total six pools representing each developmental stage for the two genotypes.

Libraries were prepared using the TruSeq SBS v5 protocol (Illumina, San Diego, CA). In particular, 10 μg of total RNA were used to isolate poly(A) mRNA after double purification of transcripts using poly(T) oligos attached with magnetic beads. Subsequent mRNA quality control was carried out on a Bioanalyzer 2100 (Agilent). Purified mRNA was fragmented using Zn-catalyzed hydrolysis and converted into double-stranded cDNA by random priming. Following end repair, single “A” base addition to 3′-end, indexed adapters were ligated and cDNA fragments of 200 ± 25 bp were purified. Purified cDNA was amplified by PCR and quality control was done by TOPO cloning and capillary sequencing. The cDNA libraries were quantified and diluted to 10 nM, after which they were multiplexed and sequenced with an Illumina HiSeq 2000 sequencer at Fasteris (Fasteris SA, Switzerland). A hundred-bp paired-end sequences were generated. Image analysis, error estimation and base calling were carried out using Illumina Pipeline (version 1.4.5) to generate the sequence data. Indexed primers were used to identify the different reads from different samples in the sequence data. Some low-quality reads were removed using a custom algorithm. Illumina TruSeq adapter sequences were clipped and the remaining reads were considered suitable for further analysis after passing quality control at Fasteris.

### cDNA sequence alignment and mapping to the reference genome

Short-read alignment and mapping of all the reads were carried on the 12x v1 annotation of the grapevine genome PN40024 [38] using BWA (Burrows Wheeler Aligner) software [39] with a maximum set of 2 mismatches in the first 32 bp sequences and a maximum of “n” mismatches in total (n from 2 to 9 depending on read length). The mapping results were processed with SAMtools [40] to extract for each transcript the number of mapped reads and determine, whether their mapping position is unique. Reads mapping to several positions on the reference sequence with the same “mapping quality” (i.e. number of mismatches and quality of the bases generating the mismatches) were attributed at random to one of them with a “0” mapping quality.

A Python script was developed to determine the distribution of mapped reads among genomic features for the wild-type and the mutant.

### Gene expression analysis

Reads mapped to multiple locations and unmapped reads were excluded from gene expression analysis. Unique reads mapping to v1_mRNA annotated transcripts were summed for each gene model and normalized by million reads (RPM) because of read coverage bias towards 3′ end of transcripts. A lower limit of detection for expression estimate was designated to be an RPM of 0.5 or, if the RPM value was less than 0.5, at least five uniquely mapped reads with identity >98% over 100 bp, as previously described by [35]. The full raw expression dataset is available at GEO under the accession number GSE58061 [http://www.ncbi.nlm.nih.gov/geo/query/acc.cgi?token=ilkdqyqehtcrraz&acc=GSE58061].

We ranked the expression of all identified transcripts by order of magnitude. Briefly, *p*-values were computed to reflect the significance of the difference between two counts (n1 and n2 corresponding to any two library combination out of the six libraries) using a binominal model. The *p*-values were log-transformed in order to allow for greater numerical stability in comparing extreme values. Next all the *p*-values and the ratios of expression between the counts were considered to compute a ranking value for each transcript (Additional file 3).

Raw uniquely mapped read counts for the wild-type and the mutant were independently subjected to differential expression (DE) analysis in a pairwise comparison between developmental stages (E-L 15 vs E-L 27, E-L 27 vs E-L 38 and E-L 15 vs E-L 38) using the software DESeq [41] in R (parameters: false discovery rate (FDR) ≤5%, log2-fold change (FC) >1). Next, DE genes were compared between the wild-type and the mutant. This strategy was preferred to the direct comparison of the two clones at each developmental stage in order to minimize the eventual differences due to asynchronous sampling.

**Figure 1:**
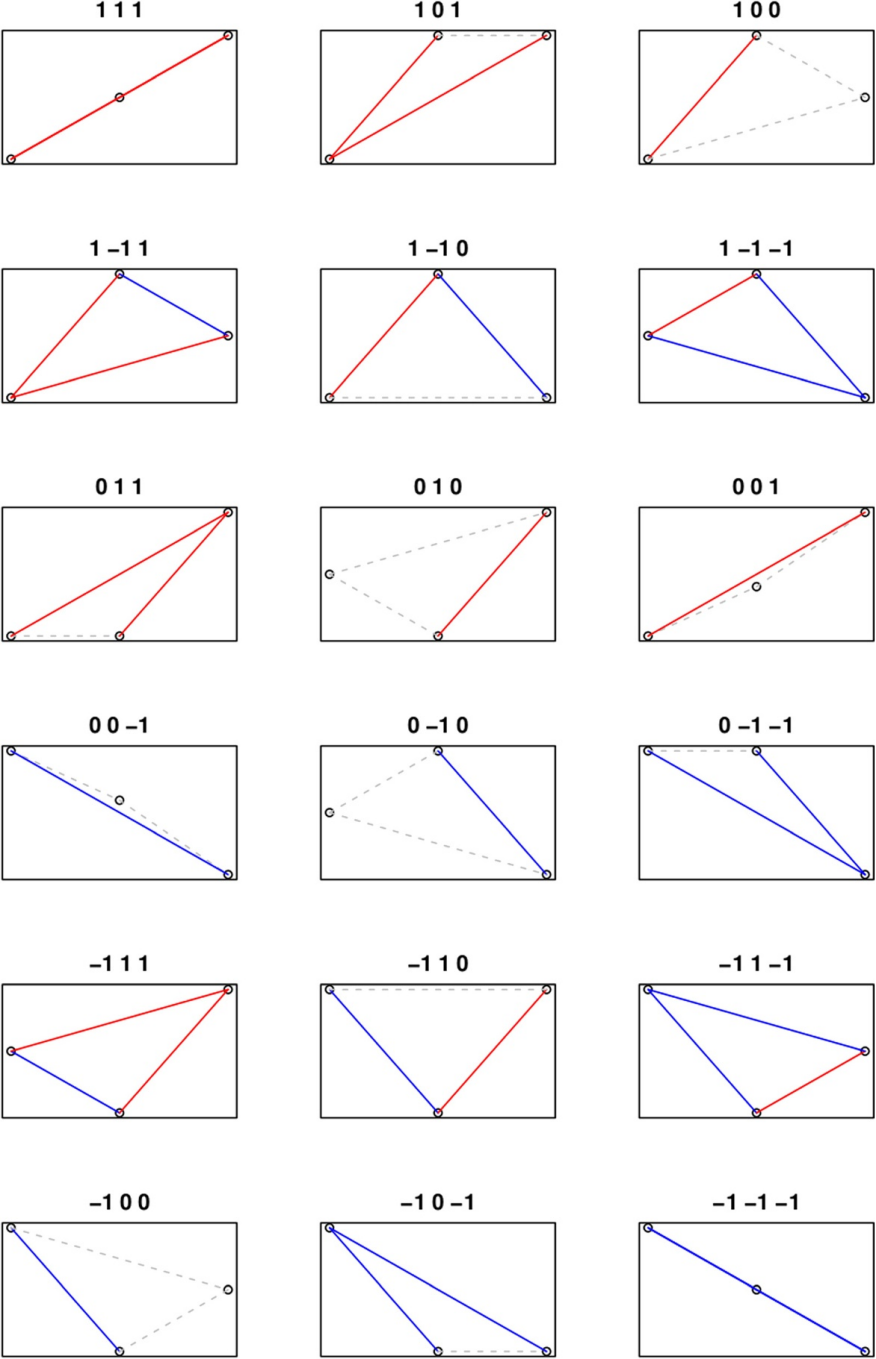
**The eighteen relevant categories of triplets of significance.** A red line indicates a significant up-regulation, a blue line a significant down-regulation, and a gray dashed line a non-significant difference between time points (the first position in the triplet corresponds to the shift from stage E-L 15 to E-L 27, the second position to the shift from stage E-L 27 to E-L 38 and the third position to the shift from stage E-L 15 to E-L 38). All DE genes for both the wild-type and the mutant formed 18 groups of differential expression patterns and the numbers of genes that fell within each group are shown in Table 3. Group 1 contains positively regulated genes along the whole time course; groups 2 and 3 consist of induced genes from stage E-L 15 to stage E-L 27, with no significant change afterwards; groups 4, 5 and 6 contain induced genes from stage E-L 15 to stage E-L 27, followed by a significant decrease in their expression; groups 7, 8 and 9 consist of stable genes from stage E-L 15 to stage E-L 27, with induced expression from stage E-L 15 or E-L 27 to stage E-L 38. Groups 10, 11 and 12 are made up of stable genes from stage E-L 15 to stage E-L 27 with reduced expression from stage E-L 15 or E-L 27 to stage E-L 38; groups 13, 14 and 15 contain repressed genes from stage E-L 15 to stage E-L 27, followed by a significant induction in their expression; groups 16 and 17 contain repressed genes from stage E-L 15 to stage E-L 27, with no significant subsequent change; group 18 contains negatively regulated genes along the whole time course.

An in-house R script was written to group DE genes with similar expression pattern based on the adjusted *p*-values. By indicating a significant up-regulation with “1”, a significant down-regulation with “-1” and a non-significant difference with “0”, the three comparisons between the developmental stages can be summarized with a triplet, e.g. “1, 0, 1”. This example indicates that there is a significant up-regulation going from the first to the second time point, no significant difference between the second and third time points, and a significant positive difference when comparing the first and last time points. Altogether, 27 different categories can be defined in this way, and 18 of these contain relevant patterns (for example the pattern “1, 1, −1” is impossible). These 18 groups are visualized in Figure 1. Each gene showing at least one significant difference between developmental stages was classified into one of these categories, for both the wild type and the mutant. The number of differentially expressed genes that fell to each pattern were compared between the wild-type and the mutant.

### Functional annotation and enrichment analysis

Wild-type and mutant genes were annotated against the v1 version of the 12x draft annotation of the grapevine genome using the CRIBI tools [42] combined with the grapevine molecular network VitisNet [43]. Next all DE genes for both genotypes were input into the AgriGO analysis tool [44]. This allowed us to identify significantly enriched gene ontology (GO) terms in the whole set of DE genes or within each group when compared with GO terms in the complete *Vitis vinifera* genome. Using a hypergeometric test, a GO term was considered significantly enriched, if the FDR was <0.05 and *p*-value <0.01 when compared to all gene transcripts annotated in the reference genome (supported in AgriGO). Further, the REVIGO web server [45] was used to summarize the processes represented in the lists of significantly enriched GO terms by removing redundant terms as described by [35].

### Selection of candidate genes

Candidate genes were chosen belonging to the three following groups:

1. Wild-type and mutant specific not DE genes, i.e. the transcripts which are expressed in the wild-type but not in the mutant and vice versa, with no significant differences between developmental stages. These genes were tested for GO annotation enrichment using AgriGO. Ultimately, genes were selected, if they fulfilled the following criteria: significant GO enrichment, RPM values above the lower limit of detection (0.5) and putative function relevant to seed development;
2. Wild-type and mutant specific DE genes, chosen based on their expression profile, fold change value, functional category enrichment, and putative function relevant to seed development. In addition, candidates were selected among DE genes with different expression profile or level of fold change in the two clones;
3. Candidate genes affecting seed content, previously identified in QTL analyses [23, 46]. These genes were compared with DE genes in the wild-type and the mutant, and the overlapping candidates were evaluated, based on their expression profile and the level of fold change.

### Real-Time PCR validation of RNA-Seq data

Quantitative real-time PCR was carried out on cDNA obtained from both biological replicates described above, one of which was used for RNA-Seq. First-strand cDNA synthesis was performed with 1 μg of total RNA in triplicate using SuperScript™ III Reverse Transcriptase (Invitrogen, Carlsbad, CA) and oligo-dT according to manufacturer’s protocol, after treatment with DNase I (Invitrogen). The transcriptional profiles of 14 genes were analyzed. SAND and GAPDH (glyceraldehyde 3-phosphate dehydrogenase) were chosen as constitutive genes for normalization after evaluation of a set of five genes with the geNorm software [47]. Their stable expression along development in the wild-type and the mutant was confirmed by RNA-Seq data. Details on gene IDs, gene annotations and primer sets are included in Additional file 4. Reactions were carried out with Platinum SYBR Green qPCR SuperMix-UDG (Invitrogen) and specific primers using the LightCycler 480 (Roche Applied Science, Mannheim, Germany). The PCR conditions were: 95°C for 5 min as initial step, followed by 50 cycles of 95°C for 15 s, 68°C for 30 s and 72°C for 10 s. Finally, a post-PCR melting curve analysis was performed to verify the specificity of cDNA amplification. Each sample was examined in three technical replicates, and analyzed using the LightCycler 480 SV1.5.0 software (Roche Applied Science). REST 2009 software was used to calculate relative expression of each gene [48].

## Results and discussion

### SSR genotyping of the wild-type and the mutant

The two clones were found to have identical allele size at all the fifty-eight analyzed microsatellite loci (Additional file 2). This result further validates the data reported by Schneider et al. [30], confirming that the mutant, usually considered to be a different cultivar, is a synonym of the wild-type.

### cDNA sequence alignment and mapping to the reference genome

Sequencing generated from 126 to 143 million and from 102 to 127 million 100-bp reads for the wild-type and the mutant, respectively (Additional file 5). After pre-processing and quality control, the majority of reads from wild-type (≈79-81%) and mutant (≈70-81%) were successfully aligned to v1_mRNA version of the 12x draft annotation of the grapevine genome [38]. A large fraction of mapped reads from each developmental stage for wild-type (≈87-89%) and mutant (≈85-87%) aligned to a single position. These uniquely mapped reads account on average for approximately 71% and 66% of the total number of sequenced reads for the wild-type and the mutant, respectively.

Distribution of mapped reads among genomic features showed that a high proportion (49% for both the wild-type and the mutant) mapped to protein coding regions indicating a high level of coverage of actual transcribed sequences (Additional file 6). The other reads mapped to splice junctions (27% and 26%), introns (14% and 16%) and untranslated regions (UTRs) (9% and 7%) for the wild-type and the mutant, respectively. The presence of intronic regions in RNA-Seq experiments is prevalent and has been attributed to various sources such as intron retention during splicing, DNA contamination during RNA-Seq preparation as well as alignment artifacts. Reads mapped to intronic regions in our data set are comparable to those obtained in similar experiments in grapevine [33]. Most of the intronic mapped reads in our data set show strand specificity hence we infer they are mainly due to unspliced mRNA in our samples and others may be due to alignment artifacts.

### Gene expression analysis

The digital, count-based nature of RNA-Seq provided a number of potential advantages for downstream data analysis and interpretation. For every gene detected in wild-type and mutant samples, uniquely mapped reads were used to generate raw expression counts and normalized expression values. The normalized expression values were calculated as RPM since it provides a useful way to assess overall expression levels between samples. Following the normalization of read counts, we analyzed the most abundant transcripts within our samples by ranking them based on their *p*-value and ratio of expression. This in turn highlighted the top most highly expressed genes across all possible pairwise comparisons of the libraries (see Methods and Additional file 7).

**Figure 2:**
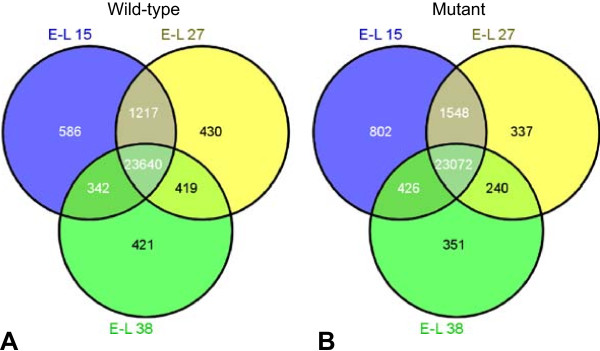
**Gene expression overlap between the three key developmental stages in wild-type and mutant plants.** Venn diagrams indicate the overlap of global gene expression signatures identified at stages E-L 15 (single flowers in compact groups), E-L 27 (young berries enlarging) and E-L 38 (berries harvest-ripe) of the wild-type **(A)** and mutant **(B)** genotypes.

Overall our data-set identified approximately 98% of grapevine annotated transcripts (representing 27,495 genes) expressed throughout the three developmental stages under study. We detected a gene expression gradient from “before flowering” to “after flowering”, i.e. for wild-type E-L 15 (25,785 expressed genes) > E-L 27 (25,706 expressed genes) > E-L 38 (24,822 expressed genes) and for mutant E-L 15 (25,848 expressed genes) > E-L 27 (25,197 expressed genes) > E-L 38 (24,089 expressed genes) (Additional file 8). To put these results into perspective, slightly more genes were expressed before fertilization in the mutant than in the wild-type and by far more genes were expressed after fertilization in the wild-type than in the mutant. In the wild-type and the mutant 23,640 and 23,072 genes were expressed in all three developmental stages, respectively (Figure 2). While it is not surprising the comparable number of genes shared by the three developmental stages in each clone, it is interesting to note that fewer genes were expressed specifically at each developmental stage: 586, 430 and 421 genes at stages E-L 15, E-L 27 and E-L 38 in the wild-type (Figure 2A) and 802, 337 and 351 genes at respective stages in the mutant (Figure 2B), which further highlights a reduction in gene expression in the mutant compared to the wild-type after fertilization. Thus we assessed what proportion of the expressed genes were common to both clones in the different stages and found that large number of expressed genes were shared among the wild-type and the mutant throughout development. In particular, 22,516 genes were commonly expressed in both clones in all three developmental stages (Table 1), 24,084 in the first two stages E-L 15 and E-L 27 (Additional file 9A) and 22,790 in the last two stages E-L 27 and E-L 38 (Additional file 9B). This was expected based on the phenotypic evaluation of the two clones that revealed similar berry development and ripening (they were at the same developmental stage in the same date). Nevertheless, a fewer number of genes were exclusively expressed in a particular developmental stage and clone (Table 1), suggesting they could be responsible for the specificity of each clone. Finally, a total of 565 genes were not expressed at all (Table 1). This set of genes could be genotype-specific and restricted to the grapevine clone PN40024 used for reference mapping.

**Table 1:** **Comparison of gene expression between the wild-type and the mutant**

	Wild-type			Mutant		
Developmental stage	E-L 15	E-L 27	E-L 38	E-L 15	E-L 27	E-L 38
Genes expressed in all developmental stages in the two clones (common genes)	22516	22516	22516	22516	22516	22516
Exclusively uniquely expressed genes for each developmental stage	183	187	169	190	70	97
Non-detected expression for each developmental stage	1145	1224	2108	1082	1733	2841
Constitutively non-expressed genes in the two clones	565	565	565	565	565	565

**Figure 3:**
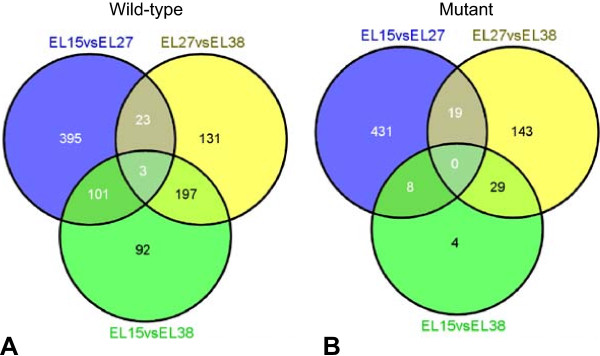
**Comparison of differential gene expression in the pairwise comparison of developmental stages in wild-type and mutant plants.** Venn diagrams indicate overlap of all differentially expressed genes obtained from each pairwise comparison between developmental stages (E-L 15 vs E-L 27, E-L 27 vs E-L 38 and E-L 15 vs E-L 38) in wild-type **(A)** and mutant **(B)**.

The results of differential gene expression analysis of RNA-Seq data in the pairwise comparison between developmental stages are shown in Figure 3. In total 1075 genes were differentially expressed (DE) in both clones. With respect to the wild-type a total of 942 genes were found to be differentially expressed during development: 522 between stages E-L 15 and E-L 27, 354 between stages E-L 27 and E-L 38 and 393 between stages E-L 15 and E-L 38 (Figure 3A). For the mutant a total of 634 DE genes were identified: 458 between stages E-L 15 and E-L 27, 191 between stages E-L 27 and E-L 38 and 41 between stages E-L 15 and E-L 38 (Figure 3B). Analysis of data set overlap (Additional file 10) revealed that about 47% of the total DE genes (501/1075) were expressed in both the wild-type and the mutant (commonly shared expression), which supports the developmental alignment of the two clones. More strikingly, the percentage of DE genes specific to the wild-type with respect to all three developmental stages is 41% (441/1075), while for the mutant it is 12% (133/1075). We further evaluated the percentage of significantly up-regulated and down-regulated genes in each pairwise comparison in both the wild-type and the mutant. On average approximately 67% of DE genes in the wild-type and 75% of DE genes in the mutant were down-regulated along development, while 33% and 25% of DE genes were induced in the wild-type and the mutant, respectively (Table 2). Taken together these results suggest that most of the expressed genes were active in different contexts along the grape berry developmental gradient (Table 1). However, significant quantitative changes occurred in individual gene expression level that corresponds to a particular stage or switch in development during seed formation. Here the mutant exhibited the strongest reduction in gene expression after fertilization (Additional file 8). It is tempting to speculate that it might be due to shut down in transcriptional processes resulting from incomplete fertilization or failure of embryo development. However, further work will be necessary to test this hypothesis.

**Table 2:** **Evaluation of significantly up- and down-regulated genes in each pairwise comparison between developmental stages**

	Wild-type						Mutant					
Pairwise comparison	E-L 27 vs E-L 15	Percentage	E-L 38 vs E-L 27	Percentage	E-L 38 vs E-L 15	Percentage	E-L 27 vs E-L 15	Percentage	E-L 38 vs E-L 27	Percentage	E-L 38 vs E-L 15	Percentage
Down-regulated genes	332	63.6	256	72.3	256	65.1	327	71.4	136	71.2	34	82.9
Up-regulated genes	190	36.4	98	27.7	137	34.9	131	28.6	55	28.8	7	17.1
Total	522		354		393		458		191		41	

Finally, we determined the expression pattern of all DE genes over the three developmental stages under investigation using the technique described in the Methods. This approach revealed transcripts from a pool of DE genes that exhibit the same patterns of expression over the three developmental stages. We present here 18 relevant groups (Figure 1). The wild-type and the mutant exhibited similar differential expression pattern except in groups 6, 10 and 18. Four main groups (3, 11, 12 and 16), accounted for about 67% of the DE genes along the three developmental stages of the wild-type. Similarly, groups 3, 11 and 16 accounted for 87% of DE genes in the mutant (Table 3). Additionally the analysis of expression pattern of all DE genes enabled us to identify relevant groups showing significant difference in the number of DE genes between the two clones, such as groups 2, 9, 10, 12 and 17 (Table 3).

**Table 3:** **Number of genes in each group of differential expression patterns for the wild-type and the mutant**

Number of groups	Gene pattern	Number of genes (Wild-type)	Number of genes (Mutant)
1	111	0	0
2	101	13	4
3	100	155	112
4	1-11	0	0
5	1-10	21	15
6	1-1-1	1	0
7	011	66	26
8	010	30	25
9	001	58	4
10	00-1	34	0
11	0-10	101	118
12	0-1-1	131	3
13	−111	0	0
14	−110	2	4
15	−11-1	0	0
16	−100	240	319
17	−101	88	4
18	−1-1-1	2	0

### Functional enrichment analysis

To assess the biological meaning of the wild-type and the mutant differential expression pattern, we examined representation of GO terms in the whole set of DE genes and within each of the eighteen groups.

When considering the whole set of DE genes the most striking difference between the two clones was the wild-type specific enrichment in GO terms related to reproduction, such as anther wall tapetum development, cell division and microsporogenesis (Additional file 11).

When considering the DE gene in each of the eighteen groups, for the wild-type we detected a number of significantly enriched GO terms in groups 3, 11, 12, 16 and 17, whereas in the mutant significantly enriched GO terms were found only in groups 11 and 16 (however, many of the GO terms in the wild-type group 17 were present in the mutant group 16) (Additional file 12). For example, we observed a specific significant enrichment of positively regulated (from stage E-L 15 to stage E-L 27) functional categories in the wild-type group 3, for which the genes were mainly related to cell wall modification. Here stage E-L 27 corresponded to “after fertilization”, a phase of berry development mainly characterized with extensive cell division. Perhaps it is likely that these genes were highly active in the wild-type and may have played important role in cell wall re-assembly to encourage cell division during seed formation and embryo development.

### Real-time PCR validation of RNA-Seq data

**Figure 4:**
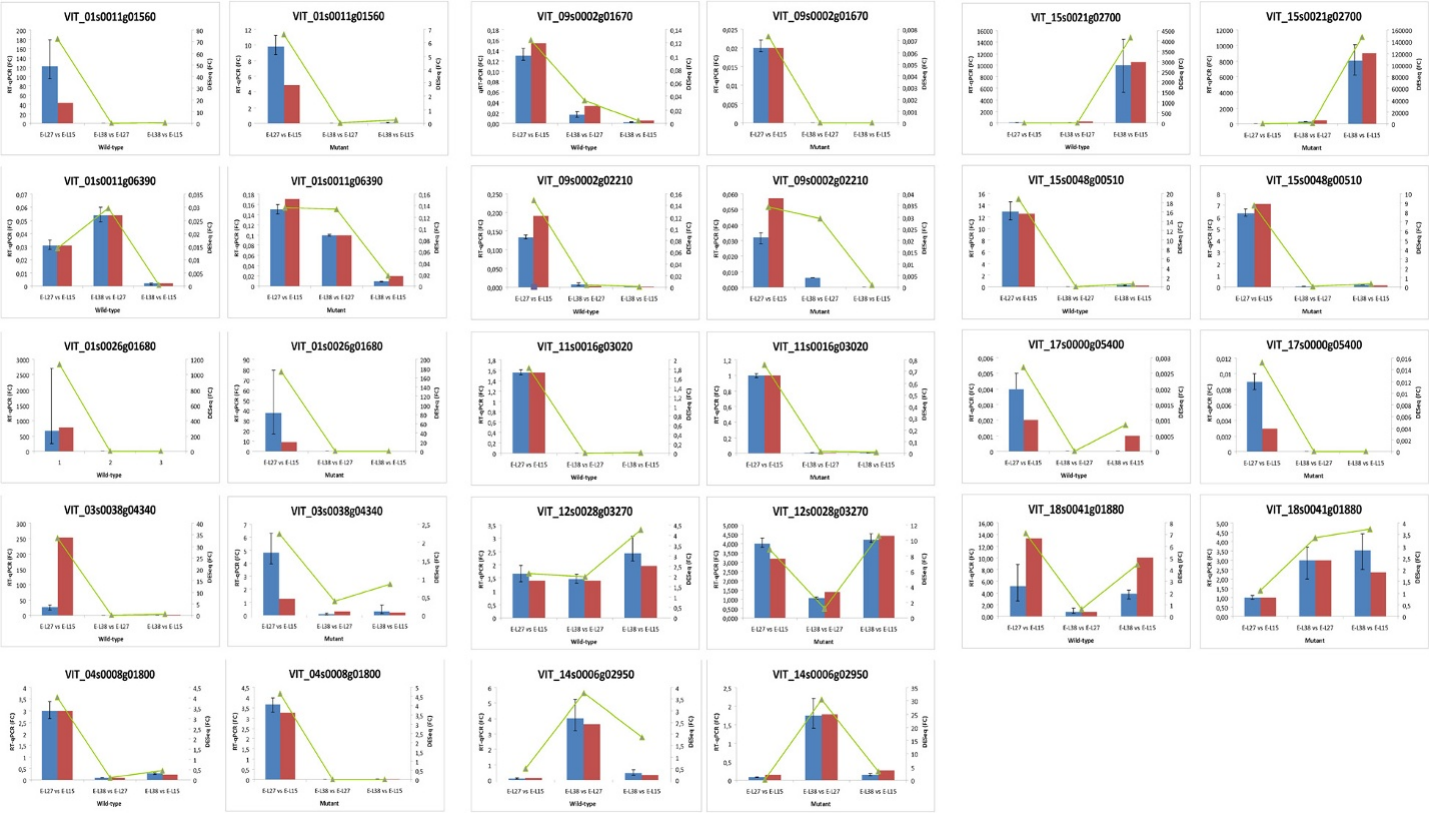
**Quantitative real-time PCR validation of RNA-Seq data.** Relative expression profile of 14 genes shows the expression fold change (FC) in the pairwise comparison between developmental stages for the wild-type and the mutant. Histograms represent expression fold changes as assessed by real-time PCR (by using REST), data are reported as means ± SE of three technical replicates (left axis). Green lines represent expression fold changes as assessed by RNA-Seq (by using DESeq, right axis). Blue column with error bar corresponds to the first biological replicate, while red column corresponds to the second biological replicate on which RNA sequencing was carried out.

To confirm the results obtained by RNA-Seq, relative expression profiles of 14 genes were analyzed by real-time PCR in the wild-type and the mutant. The tested genes encoded enzymes involved in cell wall metabolism, transcription factors from different families (MYB, MADS-box, PHD and AS2) and molecules playing a role in signaling, including hormone-mediated signaling. For both clones and all genes, the real-time PCR results were consistent with the expression profiles determined from RNA-Seq data. Seven genes had similar expression profiles in the wild-type and the mutant, while the expression of the remaining 7 genes ranged from slightly different to completely opposite which suggests that some pathways may be altered in the seedless phenotype (Figure 4). In most cases biological replicates showed a consistent expression profile.

### Selection of candidate genes

In this work gene expression analysis highlighted several genes with common and contrasting expression profiles in the two clones, which may contribute to trait variation (seed content, and the resulting berry size, are the only phenotypic differences between the two somatic variants). Therefore, in order to narrow down to specific genes whose expression and effect were altered in the seedless phenotype, we have applied the criteria described in Methods. This allowed us to select a number of candidate genes for the seedless phenotype, which are listed in Table 4 and described hereafter. Among them are genes required for fertility, cell growth and development, transcription factors and signaling molecules.

**Table 4:** **Candidate genes for seed content that have altered expression in the wild-type and the mutant**

Gene ID	Wild-type gene expression (RPM)			Mutant gene expression (RPM)			Wild-type fold change			Mutant fold change			Gene enrichment	Annotation
	E-L 15	E-L 27	E-L 38	E-L 15	E-L 27	E-L 38	E-L 27 vs E-L 15	E-L 38 vs E-L 27	E-L 38 vs E-L 15	E-L 27 vs E-L 15	E-L 38 vs E-L 27	E-L 38 vs E-L 15		
Non-DE genes specific to the wild-type														
VIT_09s0002g01980	0.5	0.7	0.2	0	0	0								Myosin-like protein XIK
VIT_15s0048g01070	0.01	0.01	1.2	0	0	0								Vacuolar iron transporter 1
VIT_04s0044g01520	0	0.8	0	0	0	0								GA 20-oxidase 2
VIT_08s0058g01200	0	0.4	0	0	0	0							sig	Alpha-expansin 2
Non-DE genes specific to the mutant														
VIT_13s0106g00290	0	0	0	0.01	0	0.1								Histone deacetylase HDA14
VIT_03s0088g00900	0	0	0	0	0.1	0.01								Pathogenesis-related protein 1B
VIT_14s0006g00050	0	0	0	0.1	0	0								Transposase, IS4
Common genes differentially regulated in wild-type and mutant														
VIT_01s0026g01680	0.02	24.6	0	0.01	1.9	0	1,133	0	nd	172.5	nd	nd	sig	Pectate lyase
VIT_05s0020g04850	1.0	2.4	89.1	0.9	0.9	101.8	nd	nd	113.1	nd	153.3	nd	sig	H1flk
VIT_15s0021g02700	0.3	7.4	1,103	0	2.0	1,396	nd	nd	4,154	inf	nd	nd	sig	Beta-expansin (EXPB4)
VIT_15s0048g00510	9.1	170.5	4.4	8.8	74.9	2.4	18.8	nd	nd	8.7	nd	nd		Pectinesterase family
VIT_15s0021g02170	631.1	3.1	0.3	81.4	0.2	0.4	0.005	nd	0.0006	0.003	nd	nd		Chalcone and stilbene synthase
VIT_18s0089g00140	40.7	0.2	2.7	3.5	0	8.0	0.004	nd	nd	0	nd	nd		1,4-beta-mannan endohydrolase
VIT_19s0015g00960	150.2	0.7	0	59.3	0.1	0.1	0.004	nd	0	0.002	nd	nd		ABC transporter G member 4
VIT_18s0001g01760	969.1	8.1	0.04	854.3	3.2	1.0	0.008	nd	0.00005	0.004	nd	nd		PISTILLATA (PI) floral homeotic protein
VIT_18s0001g13460	107.1	13.8	0	84.4	7.9	0.1	0.1	nd	0	0.1	nd	nd		MADS-box AP3
Differentially expressed genes specific to the wild-type														
VIT_01s0011g06390	29.9	0.4	0.01	3.2	0.4	0.04	0.01	nd	0.0004	nd	nd	nd	sig	MALE STERILITY 1
VIT_08s0007g07100	20.5	0.8	0.02	3.7	0.06	0.04	0.04	nd	nd	nd	nd	nd	sig	MALE STERILITY 2
VIT_07s0005g05680	17.6	0.2	3.5	4.0	0.2	0.04	0.009	nd	nd	nd	nd	nd		MALE STERILITY 5
VIT_07s0005g05720	29.3	1.6	2.3	14.5	2.6	1.2	0.06	nd	nd	nd	nd	nd		MALE STERILITY 5
VIT_15s0107g00550	172.2	19.7	21.8	111.7	26.7	2.5	0.1	nd	nd	nd	nd	nd	sig	MALE STERILITY 5
VIT_19s0014g03940	7.7	0.2	0.06	4.5	0.3	0.06	0.02	nd	nd	nd	nd	nd		SPOROCYTELESS
VIT_12s0142g00040	49.6	2.6	0.07	12.5	0.7	0.08	0.05	nd	0.002	nd	nd	nd		Glycerol-3-phosphate acyltransferase 1
VIT_00s1404g00010	17.5	22.6	0.04	18.1	14.6	0.04	nd	0.002	nd	nd	nd	nd	sig	Calmodulin-binding
VIT_01s0026g01420	22.2	58.8	0.3	22.8	57.2	0.2	nd	0.007	nd	nd	nd	nd		Wall-associated kinase 4
VIT_06s0061g00730	406.0	887.8	0.3	423.5	1,138	0.2	nd	0.0004	0.0009	nd	nd	nd		Aquaporin GAMMA-TIP3/TIP1;3
VIT_18s0001g13200	31.7	143.9	0.3	38.7	110.9	0.5	nd	0.003	nd	nd	nd	nd		Cytokinin dehydrogenase 5 precursor
VIT_05s0094g00330	0.9	16.2	969.7	0.9	4.2	712.2	17.6	nd	1,332	nd	nd	nd		Chitinase, class IV
VIT_10s0003g03030	0.02	6.5	0.4	0.06	0.5	0.6	300.4	nd	nd	nd	nd	nd		Cation/hydrogen exchanger (CHX15)
VIT_01s0011g01560	0.2	14.2	0.07	0.4	2.4	0.07	72.6	nd	nd	nd	nd	nd		TRANSPARENT TESTA16
VIT_18s0001g03010	0	1.5	0	0	0.2	0	inf	nd	nd	nd	nd	nd		BZIP transcription factor
VIT_18s0041g01880	9.5	67.4	33.2	9.8	10.7	26.7	7.1	nd	nd	nd	nd	nd	sig	MADS-box protein SEEDSTICK
VIT_03s0038g04340	0.2	6.9	0.07	0.3	0.6	0.2	33.7	nd	nd	nd	nd	nd		FERONIA receptor-like kinase
VIT_17s0000g08110	1.1	23.1	0.01	0.2	1.4	0	20.1	0.0006	nd	nd	nd	nd		Nodulin MtN3
VIT_17s0000g09000	0.8	0.4	36.3	0.5	0.009	0.04	nd	125.8	nd	nd	nd	nd		Oleosin OLE-2
VIT_07s0151g00640	12.4	5.2	453.2	17.9	3.0	1.1	nd	109.3	nd	nd	nd	nd		Globulin-1 S allele precursor
VIT_14s0128g00200	0.04	0.1	42.5	0.09	0	0	nd	540.1	1,242	nd	nd	nd		7S globulin precursor
VIT_13s0067g01250	0.06	0.1	26.0	0.02	0	0	nd	330.4	506.5	nd	nd	nd		Em protein GEA6 (EM6)
VIT_14s0108g00520	0	0.2	89.3	0.03	0.09	0.03	nd	637.6	inf	nd	nd	nd		Protease inhibitor/seed storage/lipid transfer protein (LTP)
VIT_16s0039g00220	0.4	0.2	25.2	0.3	0.1	0.7	nd	192.0	nd	nd	nd	nd		Aquaporin BETA-TIP
VIT_07s0005g05400	0	0.08	22.1	0.05	0	0	nd	360.2	inf	nd	nd	nd		ABSCISIC ACID-INSENSITIVE protein 3 (ABI3)
VIT_19s0014g04130	0.7	0.2	23.6	0.3	0.4	6.4	nd	179.8	nd	nd	nd	nd		Serine/threonine-protein kinase receptor ARK3
VIT_18s0001g01570	0.2	0.5	265.1	0.3	0.07	0.3	nd	631.3	1,548	nd	nd	nd		Seed maturation protein PM31
VIT_14s0128g00340	0.1	0.08	17.0	0.1	0	0	nd	277.3	nd	nd	nd	nd		Seed maturation protein PM34
VIT_04s0008g01610	0	0.3	176.8	0	0	0	nd	776.9	inf	nd	nd	nd		Heat shock protein 17.6 kDa class II
Differentially expressed genes specific to the mutant														
VIT_14s0219g00270	20.3	2.5	0.08	21.6	0.5	0.08	nd	nd	nd	0.03	nd	nd		TEL1 (Terminal EAR1-like 1)
VIT_12s0059g00560	14.6	1.1	1.1	17.8	0.6	0.4	nd	nd	nd	0.04	nd	nd		Fimbrin 2
VIT_04s0008g04980	15.5	2.7	0.2	10.1	0.3	0.06	nd	nd	nd	0.03	nd	nd		Boron transporter-like protein 4
VIT_09s0002g01670	9.0	1.1	0.03	11.8	0.09	0	nd	nd	nd	0.007	nd	nd		Myb domain protein 26
VIT_09s0002g01370	13.2	1.3	0.2	14.0	0.3	0	nd	nd	nd	0.02	nd	nd		AP2 AINTEGUMENTA
VIT_14s0006g02950	25.4	12.6	37.4	53.1	5.4	123.3	nd	nd	nd	0.1	nd	nd		LATERAL ORGAN BOUNDARIES protein 41
VIT_15s0046g03080	3.4	0.9	0.02	2.9	0	0	nd	nd	nd	0	nd	nd		DTA2 (downstream target of AGL15 2)
VIT_12s0134g00240	17.7	36.7	21.5	9.7	77.1	22.7	nd	nd	nd	8.1	nd	nd		Avr9/Cf-9 rapidly elicited protein 20
VIT_12s0028g03270	14.2	30.5	48.0	7.3	62.5	55.7	nd	nd	nd	8.8	nd	nd		Ethylene-responsive transcription factor 9
VIT_16s0013g00950	1.8	5.1	2.1	1.1	22.1	0.4	nd	nd	nd	20.5	nd	nd		Ethylene-responsive transcription factor ERF105
VIT_16s0013g00990	2.4	5.0	0.7	1.2	18.6	0.4	nd	nd	nd	16.1	nd	nd		Ethylene-responsive transcription factor ERF105
VIT_16s0013g01050	3.1	6.3	1.1	1.8	23.3	0.3	nd	nd	nd	13.2	nd	nd		Ethylene-responsive transcription factor ERF105
VIT_16s0013g01120	3.2	13.2	63.7	3.1	29.5	49.1	nd	nd	nd	9.8	nd	nd		Ethylene-responsive transcription factor ERF105

*Abbreviations* *: nd* not detected in a pairwise comparison, *inf* infinity (when the mean of one stage in a pairwise comparison is the denominator with value 0), *sig* significant.

#### Non-differential transcriptional processes specific to the wild-type and mutant backgrounds

### Non-DE genes specific to the wild-type

Within this category very few genes met the RPM selection criteria, however many genes were significantly enriched and some of them had a putative functional role relevant to seed development. We selected four genes that play roles in cellular process, transport and signaling.

Among cellular process genes, VIT_09s0002g01980 encodes the myosin-like protein XIK, which is involved in actin organization and biogenesis as well as actin-driven movement [43]. Among transporters, the gene VIT_15s0048g01070 encodes the vacuolar iron transporter 1 protein, implicated in iron transport and storage [43]. In seeds, iron has been demonstrated to be essential for *Arabidopsis* embryo development [49]. Among the signaling genes are VIT_04s0044g01520 and VIT_08s0058g01200. VIT_04s0044g01520 encodes GA 20-oxidase 2, which is involved in gibberellic acid biosynthesis, whereas VIT_08s0058g01200 codes for the alpha-expansin 2 protein that participates in auxin-mediated signaling pathway as well as regulating cell growth [43].

### Non-DE genes specific to the mutant

All the genes that fell within this category did not meet the RPM selection criteria described in the Methods and did not have defined function when annotated; meaning that, many of them returned no hit upon functional annotation. Nevertheless, we noticed a few genes whose functional roles could be implicated in seed development. They included the histone deacetylase *HDA14* gene (VIT_13s0106g00290), involved in chromatin organization through protein acetylation and deacetylation, a gene (VIT_03s0088g00900) coding for a pathogenesis-related protein 1B implicated in jasmonate-mediated signaling as well as in plant-pathogen interaction and a transposase *IS4* gene (VIT_14s0006g00050) that encodes a transposable element protein [43].

#### Differential regulation of common transcriptional processes in the wild-type and the mutant

Significant number of expressed genes were common among wild-type and mutant growth stages, which suggests that the corresponding proteins may function in a common pathway to carry out a wide range of developmental processes. We reasoned that many of these shared genes will respond in both clones to the same signals that control the switch from one developmental phase (before fertilization) to another (after fertilization), and will have similar pattern of expression. Indeed, differential expression analysis revealed 501 DE genes common to the wild-type and the mutant (47% of the total 1075 DE genes) (Additional file 10).

Thirty-five of these genes showed different expression between the two clones along the time course. Among the 35 genes, six were significantly enriched and three of them had a functional annotation corresponding to seed development: pectate lyase, histone H1flk-like protein (H1flk), and beta-expansin (EXPB4). Pectate lyase is an enzyme involved in cell wall organization and biogenesis by catabolizing pectin. In tomato, two pectate lyases were found to be maximally expressed at the late stage of pollen development. It was suggested that the pollen expression of these genes might relate to a requirement for pectin degradation during pollen tube growth [50]. In the present study, the pectate lyase gene VIT_01s0026g01680 was up-regulated from stage E-L 15 to stage E-L 27 in both clones but the fold change was six times higher in the wild-type compared to the mutant. Based on its functional annotation, the histone H1flk-like gene VIT_05s0020g04850 plays a role in chromatin assembly. Its *Arabidopsis* homolog encodes a P-loop containing nucleoside triphosphate hydrolases superfamily protein that functions in ATP binding activity involved in cell killing [51]. In the mutant background, this gene was specifically up-regulated from stage E-L 27 to stage E-L 38 while in the wild-type a significant differential expression with a lower fold change was observed only between stages E-L 15 and E-L 38. The beta-expansin gene VIT_15s0021g02700 was not expressed at stage E-L 15 in the mutant. Differential expression analysis in the mutant showed specific up-regulation from stage E-L 15 to stage E-L 27, in contrast to a stable expression in the wild-type between the same stages. Based on its functional annotation, this gene encodes a protein involved in auxin-mediated signaling, which implies a late induction of auxin responsive genes in the mutant.

As expected, 466 out of the 501 common DE genes shared the same group or expression profile in both the wild-type and the mutant. Functional annotation and GO term enrichment uncovered many biological processes, which included cell wall metabolism, cell cycling, primary and secondary metabolism, signaling and regulation of gene expression, water transport and abiotic stress responses. Within this set the following four genes are of interest. VIT_15s0048g00510 encodes a protein that belongs to the pectinesterase family, up-regulated from stage E-L 15 to stage E-L 27 with double fold change in the wild-type compared to the mutant. Functional annotation revealed the protein involvement in cell wall modification through pectin degradation. In *Arabidopsis*, it has been shown that cell type-specific pectin degradation is required to separate microspores during pollen development [52]. VIT_15s0021g02170, VIT_18s0089g00140 and VIT_19s0015g00960 showed a similar behavior: they were down-regulated from stage E-L 15 to stage E-L 27 in both clones, but much more expressed in the wild-type than in the mutant. VIT_15s0021g02170 encodes chalcone and stilbene synthase. Its *Arabidopsis* homolog is involved in phenylpropanoid biosynthetic process and pollen exine formation [51]. VIT_18s0089g00140 encodes 1,4-beta-mannan endohydrolase, which is implicated in fructose and mannose metabolic pathways [43]. Description of biological processes associated to its *Arabidopsis* homolog revealed a role in seed germination [51]. The *Arabidopsis* homolog of VIT_19s0015g00960 is required for male fertility and pollen exine formation as it encodes an ATP-binding cassette transporter involved in tapetal cell and pollen development [51].

Finally, within this category we identified two genes already proposed to affect seed and/or berry development [46]. They code for the PISTILLATA (PI) floral homeotic protein (VIT_18s0001g01760) and the MADS-box AP3 transcription factor (VIT_18s0001g13460). The latter co-localizes with the stable QTL for berry weight, seed number and fresh weight identified by [46].

#### Differentially expressed genes specific to the wild-type background

The 441 genes specifically modulated among the wild-type developmental stages represented 12 groups and included a range of functional categories. A large number (approximately 64%) of these genes were observed among nine groups, down-regulated from stage E-L 15 to stage E-L 27 and not differentially expressed from stage E-L 27 to stage E-L 38 or vice versa. The remaining 36% were observed in three groups, and were up-regulated in the same manner (Additional file 10).

### Down-regulated genes specific to wild-type (from stage E-L15 to stage E-L 27)

Within this category we observed several interesting genes that showed significant enrichment of GO terms and very high negative fold change. They include five genes, three of which encode similar proteins: *MALE STERILITY 1* (*MS1*, VIT_01s0011g06390), *MALE STERILITY 2* (*MS2*, VIT_08s0007g07100) and *MALE STERILITY 5* (*MS5,* VIT_07s0005g05680, VIT_07s0005g05720 and VIT_15s0107g00550). The gene coding for MS1 protein belongs to the PHD family of transcription factors. The *Arabidopsis MS1* gene was described to be a sporophytic factor controlling anther and pollen development. It plays a critical role in the induction of pollen wall and pollen coat materials in the tapetum and, ultimately, the production of viable pollen. Indeed, mutants show a semi-sterile phenotype, as their pollen degenerates after microspore release. In addition their tapetum appears abnormally vacuolated [51, 53–55]. The *MS2* gene has an unclear function in *Vitis vinifera*, however its *Arabidopsis* best match was described as a fatty acid reductase gene, involved in oxidation-reduction process and pollen exine formation [56]. The function of the *MS5* gene in *Vitis vinifera* is unknown, however in *Arabidopsis* it was suggested to be similar to *POLLENNESS3* gene [57]. Mutants of this gene in *Arabidopsis* were shown to have defects in functional microspore production that lead to the degeneration of cells within the anther locules [53]. One of the three *MS5* gene predictions co-located with a minor QTL for mean seed fresh weight on chromosome 15 [23]. The significant down-regulation of these genes from stage E-L 15 to stage E-L 27 in the wild-type implies that they were highly induced at stage E-L 15, where they exhibited maximum expression levels, perhaps to ensure viable and functional pollen development for complete fertilization. On the other hand, in the mutant, these genes were not differentially expressed. Further analysis of their RPM values in the mutant revealed very low level of expression at stage E-L 15, when compared to the wild-type. This observation might suggest abnormal pollen development in the mutant resulting in non-functional or partially sterile pollen. However, it needs to be tested and confirmed experimentally.

Within this category we found two additional genes with a putative role in ovule and pollen differentiation: *SPOROCYTELESS* (VIT_19s0014g03940) and *glycerol-3-phosphate acyltransferase 1* (VIT_12s0142g00040). The *SPOROCYTELESS* gene of *Arabidopsis* was described to encode a transcription factor that is required for the initiation of both micro- and megagametogenesis and is expressed in the sporogenous tissue of the anther and the ovule. It is involved in establishing the prospective chalaza of the ovule, plays a central role in patterning both the proximal-distal and the adaxial-abaxial axes in the ovule and regulates the anther cell differentiation. Mutant is defective in the differentiation of primary sporogenous cells into microsporocytes, and does not properly form the anther wall [51, 58, 59]. The *Arabidopsis* homolog of *glycerol-3-phosphate acyltransferase 1* gene was shown to be expressed in flower buds and siliques. Its protein is involved in metabolic processes such as phosphatidylglycerol biosynthetic process, pollen sperm cell differentiation, and response to karrikin. Interestingly, the homozygous mutant plants are male sterile [51, 60].

### Down-regulated genes specific to wild-type (from stage E-L 27 to stage E-L 38)

Within this category we observed about 30 genes with high negative fold change, the majority of which belong to the functional categories of cellular process and signaling. The most relevant for seed development appeared the genes encoding a calmodulin-binding protein (VIT_00s1404g00010), the wall-associated kinase 4 (*WAK4*, VIT_01s0026g01420), the aquaporin GAMMA-TIP3/TIP1;3 (VIT_06s0061g00730) and a precursor of cytokinin dehydrogenase (VIT_18s0001g13200). Indeed, in rice a calmodulin-binding protein was found to be essential to pollen development [61], the silencing of a member of the *WAK* family led to sterility due to anther indehiscence [62], while the aquaporin GAMMA-TIP3/TIP1;3 in *Arabidopsis* was reported to be a pollen-specific water transporter contributing to male sterility in the double knockout mutant *tip1;3/tip5;1*[63], and cytokinins were demonstrated to regulate seed yield [64].

### Up-regulated genes specific to the wild-type (from stage E-L 15 to stage E-L 27)

Amongst this group we noticed a number of genes with high positive fold change value. Besides genes encoding proteins involved in cell wall organization and biogenesis, the most relevant for seed development were found in the categories: metabolism, transport, regulation overview and signaling. For instance, we identified a chitinase class IV gene (VIT_05s0094g00330), whose best *Arabidopsis* match was described to be expressed during somatic embryogenesis in nursing cells surrounding the embryos and additionally in mature pollen and growing pollen tubes until they enter the receptive synergid [51, 65]. Among transporters, a cation/hydrogen exchanger (VIT_10s0003g03030) showed its best match with an *Arabidopsis* protein involved in pollen tube growth [51].

Of particular interest were a set of genes encoding transcription factors and signaling molecules. Among the transcription factors were TRANSPARENT TESTA 16 TT16 or AGL32 (VIT_01s0011g01560), BZIP family protein (VIT_18s0001g03010) and the MADS-box protein SEEDSTICK (VIT_18s0041g01880). The *TT16* gene encodes a MADS-box family transcription factor [43, 51]. In *Arabidopsis* it was reported to determine the identity of the endothelial layer within the ovule, to play a maternal role in fertilization and seed development and to regulate proanthocyanidin biosynthesis and cell shape of the inner-most cell layer of the seed coat [51, 66]. In canola (*Brassica napus*) it was further demonstrated that the *tt16* deficiency affects pollen tube guidance, resulting in reduced fertility and negatively impacting embryo and seed development due to the altered expression of genes involved in gynoecium and embryo development, lipid metabolism, auxin transport, and signal transduction [67]. In addition, the *TT16* gene was reported among the functional candidates potentially involved in seed and/or berry development that did not co-localize with QTLs detected for the same traits [46].

The *BZIP* gene was previously described by [68] to be expressed in pollen and other flower parts.

Although the MADS-box *SEEDSTICK* gene did not show high positive fold change, it was significantly enriched in our data. In *Arabidopsis* and rice, this gene was described to encode a MADS-box transcription factor expressed in the carpel and ovules and to play a maternal role in fertilization and seed development. Mutants indeed exhibited reduced ovule fertilization and high seed abortion [51, 69–71]. Interestingly, this gene was among those that co-localized with the stable QTLs for seed-related traits [23, 46].

The signaling molecules included FERONIA receptor-like kinase (VIT_03s0038g04340). In *Arabidopsis*, it was shown to mediate male–female interactions during pollen tube reception [72]. *Feronia* mutant had impaired fertilization because pollen tube failed to arrest by continue growth inside the female gametophyte [73]. This study concluded that female control of pollen tube reception is based on a FERONIA-dependent signaling pathway. In our investigation, we observed low expression level (0.6 RPM) of *FERONIA* receptor-like kinase gene in the mutant, compared to higher expression (6.9 RPM) in the wild-type.

Finally, within this category we identified a gene coding for a nodulin (VIT_17s0000g08110), which was up-regulated from stage E-L 15 to stage E-L 27 and down-regulated from stage E-L 27 to stage E-L 38. The *Arabidopsis* best match for this gene encodes a protein containing three domains, one of which is MtN3/saliva-related trans-membrane protein, and has function in sugar trans-membrane transporter activity [51]. In rice the genes *Xa13/Os8N3/OsSWEET11* and *Os11N3/OsSWEET14* encode proteins with two MtN3/saliva domains similar to that of *Arabidopsis*, and were identified to play important role in regulating reproductive development through promotion of fertilization. These genes were reported to have a very high expression level in rice panicles and anthers compared to other tissues. Suppressed plants showed reduced fertility or were sterile due to blockage of microspore development at the unicellular pollen grain stage. This resulted in the gradual degeneration of the immature pollen suggesting the proteins are required for pollen development in rice. In addition knockout mutants showed reduced seed size and delayed growth [74]. The significant up-regulation of the nodulin MtN3 gene from stage E-L 15 to stage E-L 27 in the wild-type compared to the mutant could imply an active role in promoting fertilization. In contrast, down-regulation of this gene from stage E-L 27 to stage E-L 38, which corresponds to a period of seed maturation (after fertilization), seems to support the notion that genes participating or promoting seed formation are tightly regulated.

### Up-regulated genes specific to the wild-type (from stage E-L 27 to stage E-L 38)

Within this category we found a gene coding for oleosin OLE-2 protein (VIT_17s0000g09000), with a putative role in oil body organization and biogenesis as well as in reproduction and seed development. Functional studies in *Arabidopsis* showed that the double mutant *ole1/ole2* had irregular enlarged oil-containing structures throughout the seed cells which led to defects in germination or seed mortality [75].

Three different genes encoded enzymes involved in primary metabolism, namely globulin-1 S allele precursor (*GLB1*, VIT_07s0151g00640), 7S globulin precursor (VIT_14s0128g00200) and Em protein GEA6 (*EM6*, VIT_13s0067g01250). Functional annotation revealed that the three genes participate in generation of metabolite precursors and serve as energy storage proteins. The maize *GLB1* gene was found to be expressed throughout embryo development specifically in seed tissues [76]. Similarly, 7S globulin precursor was described as a major storage protein in legume species [77]. In our study, the expression of the 7S globulin precursor gene was highest at wild-type stage E-L 38 while it was almost abolished in the mutant. This suggests that induction of these genes may be required to complete seed development. The best *Arabidopsis* match for the *EM6* gene was described to be the *LATE EMBRYOGENESIS ABUNDANT 6* gene, involved in response to abscisic acid, required for normal seed development, and regulating the timing of desiccation tolerance and the rate of water loss during seed maturation [51, 78].

Other interesting genes are those involved in lipid and water transport, e.g. the genes coding for a protease inhibitor/seed storage/lipid transfer protein (VIT_14s0108g00520) and aquaporin BETA-TIP (VIT_16s0039g00220).

Equally worth mentioning are two genes coding for signaling molecules, namely the abscisic acid-insensitive protein 3 ABI3 (VIT_07s0005g05400) and the serine/threonine-protein kinase receptor ARK3 (VIT_19s0014g04130). The expression of *ABI3* gene was completely abolished in the mutant from stage E-L 27 to stage E-L 38. ABI3 is a putative seed-specific transcriptional activator acting as a central regulator in ABA signaling. In different species it was described to play a major role in seed maturation and to regulate the transition between embryo maturation and early seedling development [51, 79, 80]. In *Arabidopsis* the *ARK3* gene was proposed to participate in recognition of pollen [51, 81].

Four stress response genes were also present and specifically induced, including those coding for the seed maturation proteins PM31 (VIT_18s0001g01570) and PM34 (VIT_14s0128g00340). Finally, the gene prediction for the heat shock protein 17.6 kDa class II with a putative role in protein folding (VIT_04s0008g01610) was not expressed in the mutant in all three developmental stages.

#### Differentially expressed genes specific to the mutant background

The 133 DE genes, which were peculiar to the mutant, fell within 4 groups (3, 8, 11 and 17) and were all stage specifically induced. The majority of these genes (63%) were either down-regulated from stage E-L 15 to stage E-L 27 or from stage E-L 27 to stage E-L 38, whereas 37% of them were up-regulated in the same manner (Additional file 10). The genes related to seed development showed differential expression between stages E-L 15 and E-L 27.

### Down-regulated genes specific to the mutant (from stage E-L 15 to stage E-L 27)

In this category we identified genes with high negative fold change encoding proteins with a role in cellular processes, transport and regulation of gene expression.

Among the genes involved in cellular processes we selected *TERMINAL EAR1-*like *1* (*TEL1*, VIT_14s0219g00270) and *Fimbrin 2* (VIT_12s0059g00560). The *TEL1* gene encodes an RNA binding protein with a function in shoot development, conserved among land and vascular plants [51, 82]. The *Arabidopsis* best match of *TEL1* is a member of the *MEI2*-like gene family, which plays a role in meiosis. Specific multiple mutant combinations were reported to display sterility and a range of defects in meiotic chromosome behavior [83]. The *Fimbrin 2* gene is involved in actin organization and biogenesis; its *Arabidopsis* homolog is FIMBRIN5, an actin bundling factor required for pollen germination and pollen tube growth [84]. The same function was reported in lily [85]. We observed high expression of the *TEL1* and *Fimbrin 2* genes at stage E-L 15 in both clones, however as development progressed towards stage E-L 27 a significant repression of both genes in the mutant was evident in their very low RPM values as compared to a stable expression of these genes in the wild-type. In addition the *Fimbrin2* gene in grape fell within a stable QTL for mean seed fresh weight reported by [46].

In the transport category we identified a gene encoding the boron transporter-like protein 4 (VIT_04s0008g04980). Previously, boron deficiency has been associated with the occurrence of parthenocarpic seedless grapes in some varieties of *Vitis vinifera* L [86].

We also noticed a set of genes coding for transcription factors, which included the MYB domain protein 26 MYB26 (VIT_09s0002g01670), AP2 AINTEGUMENTA (VIT_09s0002g01370) and LATERAL ORGAN BOUNDARIES protein 41 LBD41 (VIT_14s0006g02950). The *Arabidopsis* MYB26 protein was described to be involved in anther dehiscence, response to gibberellin stimulus and secondary cell wall biogenesis. Mutants for this gene produced fertile pollen but plants were sterile because anthers did not dehisce. When compared to wild type, no cellulosic secondary wall thickening was seen in the anther endothecium of the mutant [87]. The *AP2 AINTEGUMENTA* gene belongs to the *AP2 (APETALA2)/EREBP* (ethylene-responsive element binding protein) family of transcription factors, known to be key regulators of several developmental processes [88]. The *Arabidopsis* homolog was reported to have a role in ovule development among other functions. Mutants exhibited female-sterility as integuments did not develop and megasporogenesis was blocked at the tetrad stage [89]. The *LBD41* gene encodes a protein containing the conserved domain AS2/LOB. The *Arabidopsis* homolog of the *LOB* gene *ASYMMETRIC LEAVES2 (AS2)* was demonstrated to function in the repression of *KNOX* genes and in the specification of adaxial/abaxial organ polarity [90]. The maize ortholog was also reported to be required to prevent *KNOX* gene expression in lateral organs and, in addition, to promote the switch from proliferation to differentiation in the embryo sac. The failure to limit proliferation in mutant embryo sacs was shown to lead to a variety of structural defects, including the production of extra gametes and synergids. Moreover, the fertilization process was frequently abnormal, producing seeds with haploid embryos and embryos and endosperms derived from fertilization by different pollen tubes [91]. Although the role of these regulatory genes in growth and development is well documented in model species, in *Vitis vinifera* L. their specific functions are not well characterized and can only be inferred. However, we observed a general pattern in the mutant, in which expression of these genes was almost abolished at stage E-L 27 when compared to their stable expression in the wild-type.

Finally, a gene *DTA2* was observed (VIT_15s0046g03080, downstream target of AGL15). In *Arabidopsis DTA2* was reported to encode an unknown protein with no significant similarity to any known protein and to be expressed in developing seeds and in roots [92]. In our data, the *DTA2* gene from the mutant was expressed at stage E-L 15, and the expression was abolished at stages E-L 27 and E-L 38 (in contrast to the stable expression in the wild-type).

### Up-regulated genes specific to the mutant (from stage E-L 15 to stage E-L 27)

Within this category we selected six genes, one of which (VIT_12s0134g00240) encodes a signaling molecule involved in stress response. This Avr9/Cf-9 rapidly elicited protein 20 was shown to function in the initial development of the defense response in tomato [93]. The remaining five genes encode proteins involved in the ethylene-mediated signaling pathway. These are ethylene-responsive transcription factor ERF9 (VIT_12s0028g03270) and ethylene-responsive transcription factor ERF105 (VIT_16s0013g00950, VIT_16s0013g00990, VIT_16s0013g01050 and VIT_16s0013g01120). The *ERF9* gene was shown to take part in repressing the activation of pathogen related genes in *Arabidopsis*[94]. The *Arabidopsis* homolog of *ERF105* encodes a member of the ERF (ethylene response factor) subfamily B-3 of ERF/AP2 transcription factor family that is involved in processes such as regulation of transcription, respiratory burst involved in defense responses, as well as responses to mechanical stimulus and wounding [51, 94, 95]. We noticed that the expression levels of these genes were always higher at stage E-L 27 in the mutant compared to the wild-type.

It might be worthy of mention that a substantial proportion of our strongest candidate genes (that are the genes expressed specifically in either clone) were physically clustered in the vicinity of some previously identified QTLs [23, 46], mainly the loci on chromosomes 2 and 12 (Additional file 13). While there may be no causal link between their expression and trait variation, they might provide a valuable starting point for developing DNA markers linked to the target trait, as discussed in [96].

## Conclusions

At the best of our knowledge, the present study represents the first transcriptomic analysis by mRNA-Seq technology in a seeded grapevine variety and its seedless somatic variant. The examination of absolute expression count for every gene has enabled us to carry out a global investigation of gene expression at three key time-points during seed formation covering from before anthesis to after fertilization. This has in turn allowed a comprehensive description of distinguishing transcriptional events in the two lines, based on the analysis of differentially expressed genes, gene patterns and enriched functional categories. We have given a detailed account of the expression profiles of the genes potentially required to initiate and to complete seed development, including genes involved in gametophyte development, cell division, cell wall organization, as well as signaling molecules and transcription factors. Reduction in the number of transcripts observed in the mutant after fertilization seems consistent with shut down in transcriptional processes resulting from incomplete fertilization or failure of embryo development. Here the significant low expression profile of male sterility genes in the mutant and the high induction of the same genes in the wild-type suggests non-functional or partially sterile pollen in the mutant, which is in agreement with preliminary observations from the phenotypic characterization underway and encourages further investigation. We surmise that some of the candidate genes derived from this study could be useful for the development of molecular markers to assist breeding programs, especially if these genes are located in a genomic region of a known QTL for seed content. In conclusion, the data reported here have provided a rich genomic resource for functional characterization of the genes that potentially underpin seedlessness in grapevine.

### Supporting data

The data sets supporting the results of this article are included within the article and its additional files.

## Electronic supplementary material

Additional file 1: Figure S1: Sample collection. Three key time points along grape berry development corresponding to stages E-L 15 (single flowers in compact groups), E-L 27 (young berries enlarging) and E-L 38 (berries harvest-ripe) of the modified E-L system 36 were matched to the number of days from bloom (DFB) and could be assigned to two main categories: “before” (E-L 15) and “after” (E-L 27 and 38) fertilization. (A) diagram showing the match of sampling dates (expressed as E-L stages) to days from bloom in reference 4. (B) picture of the materials collected from the two lines at each sampling date. (DOCX 233 KB)

Additional file 2: Table S1: Genotypic characterization of the wild-type and the mutant. Fifty-eight SSR (simple sequence repeat) markers, spread across the nineteen chromosomes of grapevine genome, were used to genotype the wild-type and the mutant. Marker details and PCR conditions are described in Methods. Symbols: * SSR markers commonly used to discriminate grapevine varieties, - indicates homozygous or null allele. (XLSX 35 KB)

Additional file 3: Text S1: Description of the procedure adopted to rank the transcripts by order of magnitude. P-values were computed to reflect the significance of the difference between 2 counts (n1 and n2 corresponding to any two library combination out of the six libraries, independently of the genotype) using a binominal model. (DOCX 117 KB)

Additional file 4: Table S2: List of the genes analyzed in real-time PCR and primers used for their amplification. The table reports the gene IDs, gene annotations and sequences of the primer sets used to analyze the transcriptional profile of 14 genes in addition to the two constitutive genes used as reference for normalization. (XLSX 23 KB)

Additional file 5: Table S3: Summary of read mapping to v1_mRNA version of 12x grapevine genome draft annotation. Short-read alignment and mapping of all the reads were carried on the 12x v1 annotation of the grapevine genome PN40024 38 using BWA (Burrows Wheeler Aligner) software 39. The mapping results were processed with SAMtools 40 to extract for each transcript the number of mapped reads and determine, whether their mapping position is unique. Reads mapping to several positions on the reference sequence with the same “mapping quality” (i.e. number of mismatches and quality of the bases generating the mismatches) were attributed at random to one of them with a “0” mapping quality. (XLSX 11 KB)

Additional file 6: Table S4: Distribution of mapped reads among genomic features. A Python script was developed to determine the distribution of mapped reads among genomic features in the wild-type and the mutant. (XLSX 13 KB)

Additional file 7: Table S5: List of transcripts ranked by order of magnitude. P-values (or scores) were computed to reflect the significance of the difference between 2 counts (n1 and n2 corresponding to any two library combination out of the six libraries, independently of the genotype) using a binomial model. The model is described in Additional file 3. The *p*-values were log-transformed in order to allow for greater numerical stability in comparing extreme values. The sign of the *p*-value reflects the direction of the comparison (whether n1 is greater or lesser than n2). The smaller is the absolute *p*-value, the more significant is the difference between the counts. Next all the *p*-values and the ratios of expression between the counts were considered to compute a ranking value for each transcript. Afterwards the ranking values were used to sort the transcripts and show on top the biggest differences in expressions between two of the libraries. (XLSX 9 MB)

Additional file 8: Table S6: Transcript abundance measurement at each developmental stage. A lower limit of detection for expression estimate was designated to be an RPM of 0.5 or, if the RPM value was less than 0.5, at least five uniquely mapped reads with identity > 98% over 100 bp. (XLSX 11 KB)

Additional file 9: Figure S2: Gene overlap between the wild-type and the mutant in the first two and last two developmental stages. (A) Venn diagram showing shared and unique expressed genes between the wild-type and the mutant during the first two developmental stages E-L 15 and E-L 27. (B) Venn diagram showing shared and unique expressed genes between the wild-type and the mutant during the last two developmental stages E-L 27 and E-L 38. Abbreviations: WT = wild-type, MT = mutant. (PDF 146 KB)

Additional file 10: Table S7: List of all differentially expressed genes in the pairwise comparison of developmental stages in the wild-type and the mutant. Differential expression (DE) analysis was carried out on the raw uniquely mapped reads using the software DESeq with the following parameters: false discovery rate (FDR) ≤ 0.05, log2-fold change (FC) > 1. An in-house R script was written to group DE genes with similar expression pattern based on the adjusted *p*-values, such that transcripts displaying significant negative differences, significant positive differences and insignificant differences between time points were fitted to -1, 1 and 0, respectively. The first position in each triplet corresponds to the shift from stage E-L 15 to E-L 27, the second position to the shift from stage E-L 27 to E-L 38 and the third position to the shift from stage E-L 15 to E-L 38. The different sheets report the genes differentially expressed in both the wild-type and the mutant, specific to the wild-type and the mutant, respectively. Abbreviations: nd = not detected in a pairwise comparison; imp (impossible to divide) = when the basemean of one stage in a pairwise comparison is the denominator with value 0; hyphen (-) = when the basemean of one stage in a pairwise comparison is the numerator with value 0 (the corresponding logarithm doesn’t exist). (XLS 524 KB)

Additional file 11: Table S8: Functional enrichment analysis in the whole set of differentially expressed genes in the wild-type and the mutant. Using a hypergeometric test in the AgriGO toolkit, a gene ontology (GO) term was considered significantly enriched, if the false discovery rate (FDR) was < 0.05 and *p*-value < 0.01 when compared to all gene transcripts annotated in the reference genome (supported in AgriGO). The two sheets report the results of functional enrichment analysis for the differentially expressed genes in the wild-type and the mutant, respectively. Abbreviations: P = biological process, F = molecular function, C = cellular component, BG = background, Ref = reference. Asterisks indicate terms commonly enriched in the wild-type and the mutant. (XLSX 61 KB)

Additional file 12: Table S9: Functional enrichment analysis within groups of differentially expressed genes in the wild-type and the mutant. Using a hypergeometric test in the AgriGO toolkit, a gene ontology (GO) term was considered significantly enriched, if the false discovery rate (FDR) was < 0.05 and *p*-value < 0.01 when compared to all gene transcripts annotated in the reference genome (supported in AgriGO). The different sheets report the results of functional enrichment analysis within specific clusters of differentially expressed genes in the wild-type and the mutant. Abbreviations: P = biological process, F = molecular function, C = cellular component, BG = background, Ref = reference. Asterisks indicate terms commonly enriched in the wild-type and the mutant clusters 11 and 16. (XLSX 182 KB)

Additional file 13: Table S10: Proportion of RNA-Seq-derived candidate genes in the physical proximity of seed-related QTLs. Genes that were specifically expressed in either clone (with or without significant modulation between developmental stages) were mapped to each of eight chromosomes carrying seed-related QTLs, identified previously by [23, 46]. The proportion of genes falling within a 10-Mb region centered on each QTL peak was calculated, as described in [96]. Abbreviations: chr = chromosome. (XLSX 9 KB)

## Abbreviations

bp: Base pair
cDNA: Complementary DNA
cv: Cultivar
DE: Differentially expressed
DFB: Days from bloom
DNA: Deoxyribonucleic acid
dNTP: Deoxyribonucleotide triphosphate
FC: Fold change
FDR: False discovery rate
GO: Gene ontology
mRNA-Seq: Messenger ribonucleic acid sequencing
MT: Mutant
QTL: Quantitative trait locus
RPM: Reads per million
qRT-PCR: Real time reverse transcription polymerase chain reaction
SE: Standard error
SSR: Simple sequence repeat
UTR: Untranslated region
v1: Version 1
WT: Wild-type.
