# Supplementary material for: Transcriptome analysis during berry development provides insights into co-regulated and altered gene expression between a seeded wine grape variety and its seedless somatic variant
Source: BMC Genomics. 2014 Nov 27;15(1):1030. doi: 10.1186/1471-2164-15-1030 (PMC4301461; doi:10.1186/1471-2164-15-1030)
Supplement: Supplementary file 1 — Additional file 1: Figure S1: Sample collection. Three key time points along grape berry development corresponding to stages E-L 15 (single flowers in compact groups), E-L 27 (young berries enlarging) and E-L 38 (berries harvest-ripe) of the modified E-L system 36 were matched to the number of days from bloom (DFB) and could be assigned to two main categories: “before” (E-L 15) and “after” (E-L 27 and 38) fertilization. (A) diagram showing the match of sampling dates (expressed as E-L stages) to days from bloom in reference 4. (B) picture of the materials collected from the two lines at each sampling date. (DOCX 233 KB) [file 12864_2014_6843_MOESM1_ESM.docx]

**Figure S1. Sample collection.**


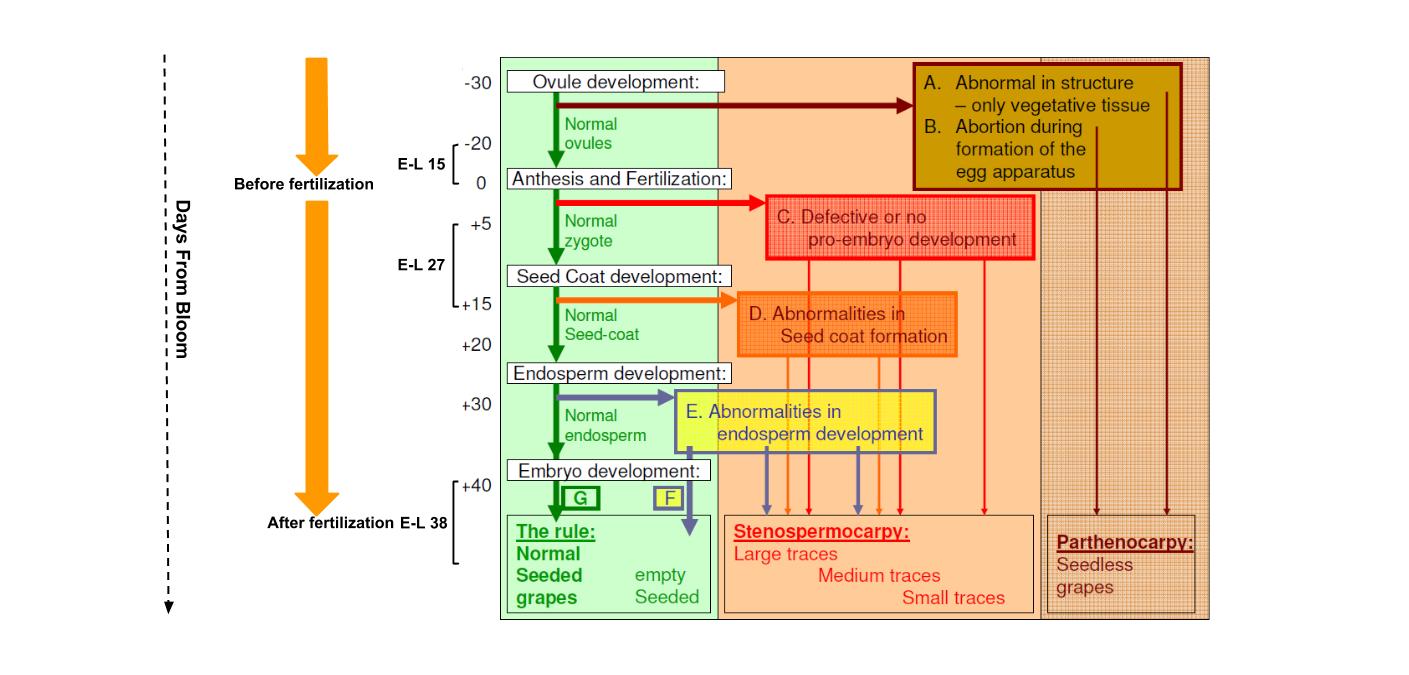


(A) diagram showing the match of sampling dates (expressed as E-L stages) to days from bloom in reference 4.


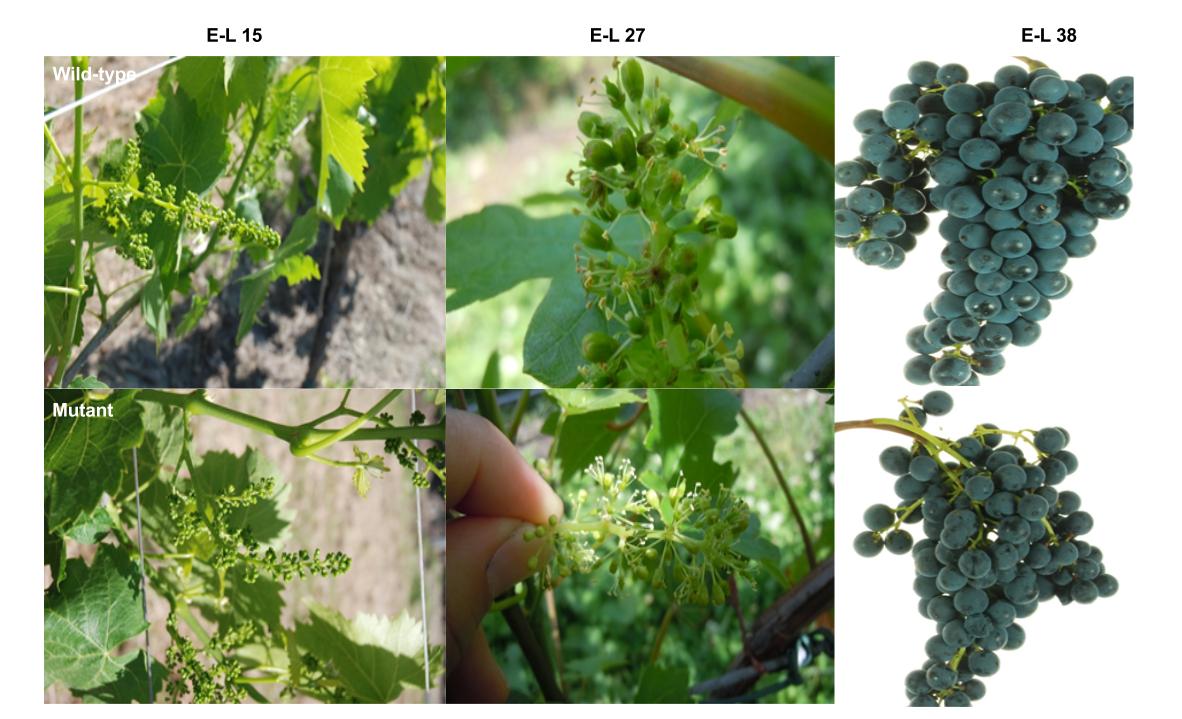


(B) picture of the materials collected from the two lines at each sampling date.

In 2009 anthesis was recorded for both lines between the last week of May and the first week of June (personal communication). With this prior knowledge of expected date of anthesis, RNA-Seq sampling for 2010 season was planned such that the initial sample collection will fall between 0 and 30 days before bloom in order to create an inventory of gene expression before fertilization (or flowering). For successive inventories of gene expression after fertilization, samplings were planned to take place between 0 and 15 days post anthesis and subsequently at harvest to cover the whole ripening process. Using the E-L system as a guide (see reference 36), the first sampling was done on 12th May 2010 when 8 leaves were physically observed to be clearly separated and single flowers were in compact groups (corresponding to the stage E-L 15), and fell between 15 and 20 days from the expected date of full bloom. The same was done for the stage E-L 27, when the date of sampling (10th June 2010) was discounted from the actual date anthesis commenced, and it fell between 0 and 10 days post anthesis. The last sampling date (16th September 2010) corresponded to the stage E-L 38 (harvest), which was more than 40 days post anthesis. Note: anthesis in 2010 was observed between 31st May and 3rd June.
