# Supplementary material for: Transcriptome analysis during berry development provides insights into co-regulated and altered gene expression between a seeded wine grape variety and its seedless somatic variant
Source: BMC Genomics. 2014 Nov 27;15(1):1030. doi: 10.1186/1471-2164-15-1030 (PMC4301461; doi:10.1186/1471-2164-15-1030)
Supplement: Supplementary file 3 — Additional file 3: Text S1: Description of the procedure adopted to rank the transcripts by order of magnitude. P-values were computed to reflect the significance of the difference between 2 counts (n1 and n2 corresponding to any two library combination out of the six libraries, independently of the genotype) using a binominal model. (DOCX 117 KB) [file 12864_2014_6843_MOESM3_ESM.docx]

**Text S1. Description of the procedure adopted to rank the transcripts by order of magnitude.**

*P*-values (or scores) were computed to reflect the significance of the difference between 2 counts (n1 and n2 corresponding to any two library combination out of the six libraries, independently of the genotype) using a binomial model. The model is described below. The *p*-values were log-transformed in order to allow for greater numerical stability in comparing extreme values. The sign of the *p*-value reflects the direction of the comparison (whether n1 is greater or lesser than n2). The smaller is the absolute *p*-value, the more significant is the difference between the counts. Next all the *p*-values and the ratios of expression between the counts were considered to compute a ranking value for each transcript. Afterwards the ranking values were used to sort the transcripts and show on top the biggest differences in expressions between two of the libraries.

**Model description**

Assuming we sequenced N1 reads in sample1 (resp N2 reads in sample2), and n1 of those reads (resp. n2) are mapping into a given region of interest in the genome, we are interested in determining whether the expression in sample1 is significantly different from the expression in sample2. If we assume the events have the same probability of been observed in the two samples, n1 and n2 should follow a binomial distribution with the same probability of event *p*=(n1/N1 + n2/N2)/2. We can then estimate the probability of observing a count less than n1 or greater than n2 according to this model. Furthermore, we can put a sign on the probability to reflect the direction of the comparison. For example, a score of -0.9 may be interpreted as: «there is 90% chance that sample1 is under-expressed relatively to sample2»; and a score of +0.9 may be interpreted as «there is 90% chance that sample1 is over-expressed relatively to sample2». The picture below shows an overview of the score obtained when n1 and n2 are between 0 and 100, and N1, N2 are fixed to 1'000'000. We can for example see that under this model there is 95% probability that a count of n1=20 compared to a count of n2=40 is significantly different when there are 1'000'000 events in each sample. Note: We have observed that the model is not very well appropriate to compare large values. This issue may be related to a saturation effect.

Overview of the scores obtained with the binomial model when comparing two counts (n1, n2) between 0 and 100 with (N1,N2) fixed to 1'000'000.
