# Supplementary figures and images for: Transcriptome analysis during berry development provides insights into co-regulated and altered gene expression between a seeded wine grape variety and its seedless somatic variant
Source: BMC Genomics. 2014 Nov 27;15(1):1030. doi: 10.1186/1471-2164-15-1030 (PMC4301461; doi:10.1186/1471-2164-15-1030)

Wild-type and Mutant E-L 15 & 27

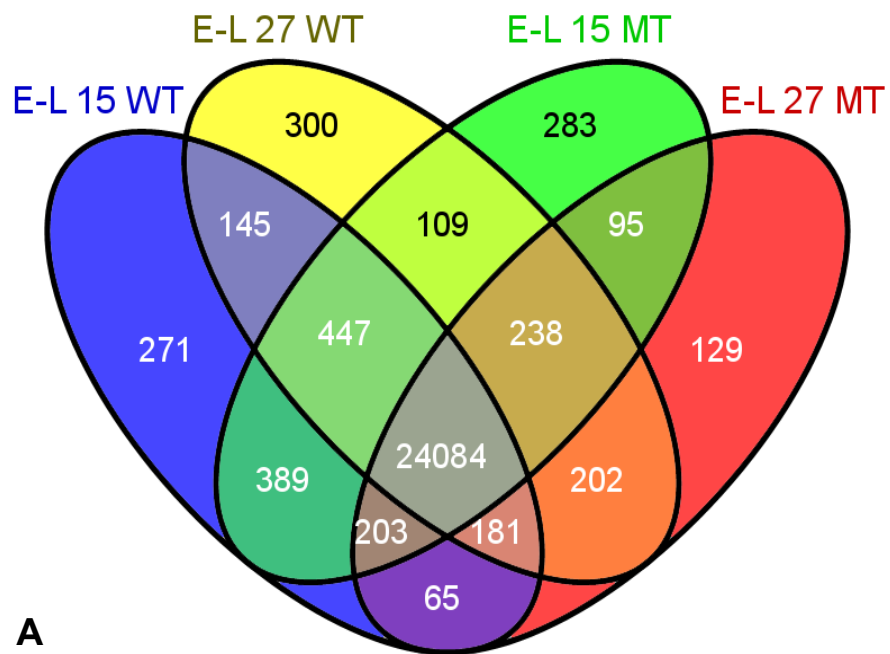

Wild-type and Mutant E-L 27 & 38

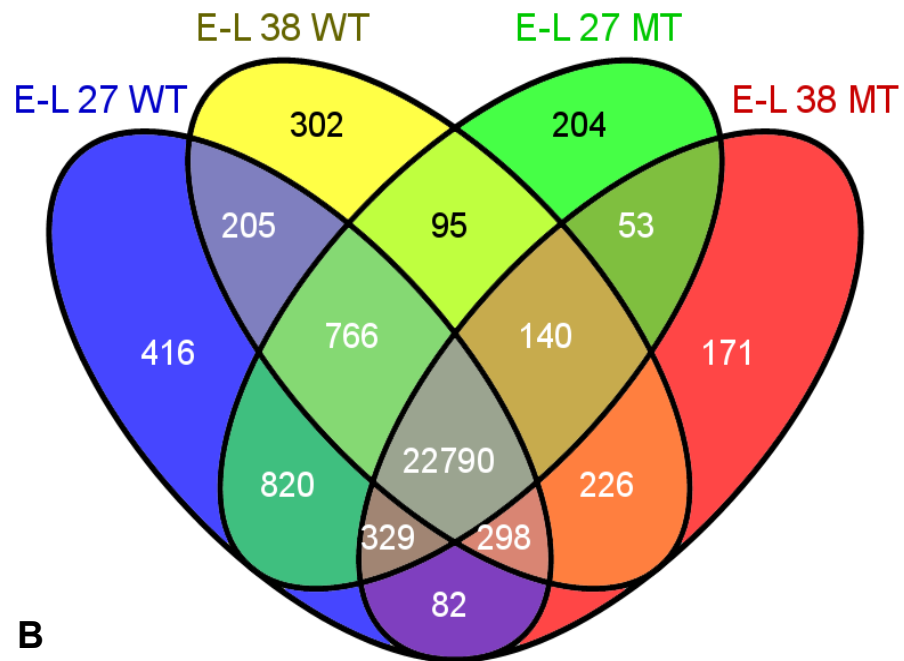

Supplement: Supplementary file 9 — Additional file 9: Figure S2: Gene overlap between the wild-type and the mutant in the first two and last two developmental stages. (A) Venn diagram showing shared and unique expressed genes between the wild-type and the mutant during the first two developmental stages E-L 15 and E-L 27. (B) Venn diagram showing shared and unique expressed genes between the wild-type and the mutant during the last two developmental stages E-L 27 and E-L 38. Abbreviations: WT = wild-type, MT = mutant. (PDF 146 KB) [file 12864_2014_6843_MOESM9_ESM.pdf]
